# Src Family Kinases as Therapeutic Targets in Advanced Solid Tumors: What We Have Learned So Far

**DOI:** 10.3390/cancers12061448

**Published:** 2020-06-02

**Authors:** Stefano Martellucci, Letizia Clementi, Samantha Sabetta, Vincenzo Mattei, Lorenzo Botta, Adriano Angelucci

**Affiliations:** 1 Department of Biotechnological and Applied Clinical Sciences, University of L’Aquila,67100 L’Aquila, Italy; s.martellucci@sabinauniversitas.it (S.M.); letizia.clementi@graduate.univaq.it (L.C.); samantha.sabetta94@libero.it (S.S.); 2 Biomedicine and Advanced Technologies Rieti Center “Sabina Universitas”, 02100 Rieti, Italy; v.mattei@sabinauniversitas.it; 3 Department of Biological and Ecological Science, University of Tuscia, 01100 Viterbo, Italy; lor.botta83@gmail.com

**Keywords:** Src, src family tyrosine kinases, cancer metastasization, cancer migration, cancer invasion, epithelial-to-mesenchymal transition, clinical trial, targeted therapy, dasatinib

## Abstract

Src is the prototypal member of Src Family tyrosine Kinases (SFKs), a large non-receptor kinase class that controls multiple signaling pathways in animal cells. SFKs activation is necessary for the mitogenic signal from many growth factors, but also for the acquisition of migratory and invasive phenotype. Indeed, oncogenic activation of SFKs has been demonstrated to play an important role in solid cancers; promoting tumor growth and formation of distant metastases. Several drugs targeting SFKs have been developed and tested in preclinical models and many of them have successfully reached clinical use in hematologic cancers. Although in solid tumors SFKs inhibitors have consistently confirmed their ability in blocking cancer cell progression in several experimental models; their utilization in clinical trials has unveiled unexpected complications against an effective utilization in patients. In this review, we summarize basic molecular mechanisms involving SFKs in cancer spreading and metastasization; and discuss preclinical and clinical data highlighting the main challenges for their future application as therapeutic targets in solid cancer progression

## 1. Introduction

Non-receptor tyrosine kinases represent a large cytosolic enzyme family, the most representative of which in mammals is the Src Family tyrosine Kinases (SFKs), including Src, the first ever described tyrosine kinase proto-oncogene. To date tyrosine kinases (TKs) represent also the most representative class of targeted proteins in anticancer therapy [1]. Ten additional kinases with homology to Src have been identified: Blk (B-lymphoid tyrosine kinase), Fgr (gardner-rasheed feline sarcoma), Fyn (proto-oncogene tyrosine-protein kinase Fyn), Frk (Fyn-related kinase), Hck (hematopoietic cell kinase), Lck (lymphocyte specific kinase), Lyn (tyrosine-protein kinase Lyn), Yes (yamaguchi sarcoma), Yrk (Yes-related kinase), and Srms (Src-related kinase lacking C-terminal regulatory tyrosine and N-terminal myristylation sites) [2,3,4].

Although several studies have demonstrated the presence of some functional redundancy between co-expressed SFKs, there is also plenty of evidence for non-overlapping functions. However, of the 11 SFK members, Src, Fyn, and Yes have been most frequently implicated in tumorigenesis and metastasis formation [5]. Indeed, Src, Fyn, and Yes, but also Frk, are widely expressed in a variety of tissues, whereas for the other members the protein expression is more tissue-restricted with a prevalence in cells of hematopoietic origin. Nonetheless, in contrast to the widely characterized agonistic role of SFKs in cancer, noteworthy specific examples of antagonistic role have been reported. Frk was described as tumor suppressor in different cancers, at least partly by protecting the tumor suppressor Phosphatase and TENsin homolog (PTEN) from degradation [6]. However, other reports of a potential pro-oncogenic function of Frk also exist, such as in studies evaluating the therapeutic potential of SFKs in liver and pancreatic cancer cell lines [7,8]. At status quo, the cellular roles of SFKs, specifically in the context of cell proliferation and invasion, should be evaluated on a tissue-specific basis and identification and characterization their cellular substrates will be helpful in deciphering the context-specific function of SFKs.

Yrk has been described only in adult chicken and it was detected in hematopoietic cells, cerebellum, spleen, lung, and skin [9,10]. In solid tumors, an increased expression of many members of the family was generally observed, and also for those SFKs with a prevalent expression in normal hematological cells, a de novo presence was frequently reported in non-hematological cancer tissues (Table 1) [11,12].

Elevated protein levels were shown for Src, Frk, Lyn, Blk, Hck, and Srms, in different tumors, with Yes that demonstrated the highest number of positive cases in a variety of tumors.

SFKs interact directly with several tyrosine kinase receptors, G-protein-coupled receptors, steroid receptors, signal transducers, and activators of transcription, leading to a diverse array of biological functions from cell survival to metastases [13]. The importance of SFKs in metastatic spreading is a consolidated evidence and it has been associated with different mechanisms including the promotion of tumor cell motility, epithelial-to-mesenchymal transition (EMT) as well as adaptation of resident cells in the secondary microenvironment [14,15].

Specifically, Src overexpression or overactivation was associated with the aberrant formation of invadopodia, critical morphological structures employed by cancer cells to intravasate into the bloodstream and extravasate into secondary sites during the metastatic process [16,17].

In addition, Src is a potential target in tumor associated cells. In fact, Src kinase inhibition counteracts those mechanisms underlining some tumor-induced phenotypic switches, including stimulation of cancer associated fibroblasts (CAFs), angiogenesis, and immune infiltrate [18,19,20]. Although Src is ubiquitously expressed, the primary phenotype associated with mutant Src−/− mice is osteopetrosis, a condition caused by the failure to resorb bone. This phenotype results from the blockade of osteoclast maturation and in particular from the inhibition of actin dynamics and its organization, a phenomenon essential in resorbing bone [21]. These data have designated Src as an attractive therapeutic target for the prevention and treatment of bone metastases [22].

## 2. SFKs Structure and Regulation

SFKs display large sequence and structural similarity. The most variable long sequence among SFKs is localized in the “unique domain” connecting the SH3 and SH4 domains, but to which has been assigned specific regulatory role only in few cases [23]. Src, the prototype kinase of SFKs, is a 60-kDa protein able to associate with the plasma, perinuclear, and endosomal membrane via a N-terminal anchoring region that includes the SH4 domain, rich in positively charged residues [24]. During intense cytoskeleton dynamic and cell migration, SFKs are targeted to focal adhesions (FAs), a phenomenon that is regulated mainly by the SH2 domain [25]. Recent studies have reported the presence of Src also in the nuclear compartment, and this localization seems to be relevant in the progression of some solid tumors [26,27]. While the Src enzymatic activity is localized in the SH1 domain, the SH2 and SH3 domain through intra-protein and protein–protein interaction can control the catalytic activity and the recognition of substrates. In fact, protein–protein interaction and Tyr phosphorylation status determine the existence of a dynamic equilibrium between closed-inactive and open-active form of SFKs. The open conformation allows the phosphorylation of Tyr419 (in human Src) in the activation cycle, which improves catalytic activity. In the canonical pathway, Src activation is initiated by dephosphorylation of pTyr530 (in human Src) followed by a conformational change that permits autophosphorylation at Tyr419 with full activation [28].

In all SFKs an important activation control is exerted by phosphorylation status in C-terminal Tyr residues (Tyr530 in human Src). The phosphorylation of Tyr530 determines conformational changes leading to intramolecular interaction with the SH2 domain and inactivating kinase activity [29]. The close association between spatial conformation and kinase activation, obligates researchers to carefully consider the interacting ability of SFKs with other proteins in critical cell compartments. The SH3 domain, consisting of 50–60 amino acids, binds proline rich sequences and it can interact with the linker domain (PPII-linker) on the back of the catalytic domain, thus promoting a "closed" conformation that prevents interaction with substrates. Several ligands are known to compete with the SH3/PPII-linker interaction: the progesterone receptor, p130Cas, AFAP-110, and Nef. The SH2 domain, consisting of about 100 amino acid residues, binds phosphorylated Tyr residues in C-terminal domain in the inactive conformation or, in the active form, on other proteins [30]. In the open form, the inhibitory tyrosine residues are accessible to phosphatase and dephosphorylation coincides with the destruction of all intramolecular interactions and the phosphorylation of the activating site that induces a complete kinase activation. Phosphorylation of pTyr530 works in conjunction with dephosphorylation of pTyr419, mediated by proline-enriched tyrosine phosphatase (PEP), in allowing the complete inactivation of SFKs in vivo [31]. However, the binding with external ligands still allows SFKs to open its spatial conformation and to remain active even in the presence of terminal C-tail phosphorylation. This indicates that phosphorylation of inhibitory tyrosine and the resulting intramolecular interaction are not sufficient to maintain SFK activity in a complete inhibited status in vivo [32]. In agreement with a context-dependent activation model, experimental evidence suggests that the activated phosphatase CD45 can also function as a negative regulator of SFK by dephosphorylating pTyr419 [33].

During tumor progression, Src activity becomes abnormally high and since activating mutations or amplification are very rare in human Src, an altered extrinsic control of phosphorylation by kinase or phosphatase and of interacting partners may represent an important mechanism for activation of Src. The phosphorylation of Tyr530 is determined by the action of different tyrosine kinases including Csk (C-terminal Src kinase) and Chk (Csk-homologous kinase) [34]. Csk kinase acts as the main negative regulator of Src and under basal conditions in vivo, 90–95% of Src is phosphorylated in Tyr530. The importance of phosphorylation of Tyr530 in the mechanism of inhibition of Src kinase activity is suggested by experimental evidence in which knockout mutant shows a constitutive basal activity associated with oncogenic transformation [35]. Several tyrosine phosphatases (PTPs) are involved in terminal C-tail dephosphorylation mechanism. Their role in SFK regulation is complex showing tumor specificity and specific roles for the different members of the kinase family.

PTP1B phosphatase, which is upregulated in many human solid tumors as colon, lung, and breast cancer, is responsible for the dephosphorylation of Tyr530 and subsequent activation of Src and therefore, for increased tumorigenicity [36,37]. CD45, abundantly expressed in all nucleated cells of hematopoietic origin, is the most important phosphatase activating SFKs by dephosphorylation of the terminal C-tail [38]. In contrast to Lck and Fyn, the Src C-terminal phosphotyrosine does not appear subject to dephosphorylation by CD45 [39]. SHP-1 and SHP-2 are two tyrosine phosphatases localized in cytosol. SHP-1 is primarily present in cells of hematopoietic origin, SHP-2 is ubiquitous and experimental evidence has established that SHP-1 is involved in dephosphorylating and activation of Src [40]. However, SHP-1 and CD45 are also able to dephosphorylate Tyr-394 in the catalytic domain of Lck and to inactivate Lck, but not Src, suggesting that these phosphatases are not involved only in activation of SFKs but also in the modulation of their specificity [41,42]. SHP-2 can regulate SFKs activity in several ways: by an enzymatic mechanism involving terminal C-tail dephosphorylation, and a non-enzymatic mechanism linked to the control of Csk recruitment to the plasma membrane. In this case, SHP-2 catalyzes the dephosphorylation of the PAG protein (phosphoprotein associated with glycosphingolipid-enriched microdomains), which in turn are unable to recruit Csk. Thus, the activity of SHP-2 can result in the failure to activate the inhibitory control of Csk in membrane-associated complexes containing SFKs [43].

## 3. SFKs in Cancer Signal Transduction, Migration, and Invasion

Since the original identification of a transmissible agent responsible for the development of solid tumors in chickens, now known to be a retrovirus encoding the v-SRC, significant progress has been made in defining the functions of its human homolog, Src [44]. One of the aspects associated with oncogenic potential that emerged early on from the studies, was the ability of SFKs to stimulate cell motility. The relationship between Src activation and cancer invasion, the most advanced phase of solid tumor progression, appears to be significant in a wide number of preclinical human cancer models, thus prompting the optimistic use of SFK inhibitors in clinical trials [45]. The SH3 domain of SFKs permits the association with actin filaments that guide the translocation of SFKs towards the cell periphery where they can interact with other molecular partners allowing two major transduction events: (i) signaling from growth factor receptors, which mainly affects cell growth, (ii) signaling from adhesion receptors including integrins and E-cadherin, which mainly regulate the functions of the cytoskeleton [46]. To date, most evidence suggests that Src has a predominant role in the maintenance of the invasive phenotype, rather than of the cell cycle progression [23]. FA junctions, that are key structure in regulating cell motility, are formed by the docking activity of SFKs, through the accessibility of SH2 domain.

FAs are protein complexes that play a critical role in dynamically controlling the coupling of actin filaments and the extracellular matrix. During migration, integrins, heterodimeric transmembrane receptors bind directly to the extracellular matrix (ECM) on the outside of the cell and link ECM to the actin cytoskeleton through FA. In turn, FAs contain signaling proteins that regulate cytoskeletal dynamics, including changes in actinomyosin contractility. The formation of FAs is dependent on the phosphorylation status and the presence of docking sites in participating proteins. SFKs can regulate the phosphorylation status of many substrates in the FA and participate directly in the formation of the complex through the docking site SH2. Src associates with FA in FAK-dependent manner. In addition, the association of Src with FAs plays a key role in transducing Src-mediated signals, in the promotion of FA formation/maturation and in enhancing cell migration [25]. Src substrates in FA include FAK, p130Cas, tensins, paxillin, and crk [47,48]. Src and FAK in their activated states, form a functional bipartite kinase complex that is fundamental in the signal transduction and in formation of lamellipodia. In fact, phosphorylation of p130Cas by the FAK/Src complex provides a binding site for the SH2 domain of CrkII, which in turn serves as a docking site for Rac1 [49]. Involvement in FA activity has been described also for other members of SFKs including Fyn [50] and Yes, and through the use of deleted-mutant SFY (src-/-fyn-/-yes-/-) it was demonstrated a strong dependence of cancer cell migration and metastatic ability on these kinases [51]. Invadosomes, which include invadopodia, are F-actin based structures used by cancer cells to interact with and to degrade ECM. These structures were initially discovered in v-Src transformed chicken fibroblasts. Src may target different actin-binding proteins during distinct stages of invadosome formation and maturation to regulate actin dynamics and invadosome function. Various signaling pathways have been shown to be involved in the formation of invadosomes, resulting in focally releasing of metalloproteases (MMPs) that degrade ECM. During invadosome precursor formation, signaling proteins such as transmembrane growth factor receptors and/or SFKs organize with structural and adaptor proteins including Tks5, Nck1, and cortactin to recruit the Arp2/3 complex and mediate actin polymerization [52]. Cortactin is a multi-functional Src substrate that is involved in both the assembly and maturation of invadosomes and is important for invasive cell migration [53,54]. Src phosphorylates cortactin at three sites, Y421, Y466, and Y482, and cortactin phosphorylation is important for the formation of invadopodia [55]. In addition to its effects on the actin-severing activity of cofilin, phosphorylated cortactin facilitates actin polymerization through its interactions with N-WASP and Arp2/3 [56,57]. MAbp1, in contrast to cortactin, impairs invadopodia formation and invasive cell migration and it is phosphorylated by Src at two sites, Y337 and Y347 [58]. The spatiotemporal phosphorylation of cortactin and mAbp1 can regulate invadosome dynamics, with cortactin present at invadopodia during early assembly and stabilization, while mAbp1 is present at mature podosome dots [58,59]. Src is necessary also during invadosome disassembly. Src phosphorylates paxillin, which through Erk signaling, activates the protease Calpain-2, which in turn cleaves multiple invadosome proteins including paxillin and cortactin.

Migration and invasion are acquired abilities in epithelial tumor cells and require the disassembly of adherent and tight-junctions and the inhibition of anoikis. These latter transformations are early steps in the EMT suggesting a role for SFKs in this phenomenon. Importantly, Src plays a control role in the expression of E-cadherin. Src inhibition induces in breast cancer cells a reverted epithelial phenotype associated with an increase of E-cadherin and a decrease of vimentin and it blocks cancer cell migration [60]. Oncogenic expression of Src in pancreatic tumor cells was also associated with a reduced expression of E-cadherin in favor of vimentin [61]. Similar results were obtained also in other tumors including prostate and renal cancer [62]. Given the involvement of SFKs activity in the EMT, cancer migration and invasion, Src family represents an attractive target for advanced stages of cancer progression.

## 4. SFKs and Cancer Progression

SFKs are frequently overexpressed and/or aberrantly activated in a variety of epithelial and non-epithelial cancers. Further, the extent of increased SFK activity often correlates with malignant potential and patient survival; in fact, it was observed in several tumors, that SFKs expression was associated with cancer stage, cellular differentiation, and formation of distant metastases.

### 4.1. Breast Cancer

Available clinical data indicate that breast cancer has a high spreading ability with the metastatic process that starts long before the diagnosis of metastases: the elevated number of occult metastases revealed in an autopsy series of breast cancer patients confirmed this hypothesis. The constitutive activation of tyrosine kinases of the epidermal growth factor receptor (EGFR) family, and of the downstream signaling pathway, was suggested as a key step in supporting the aggressive phenotype of breast cancer cells.

Src expression is detectable in almost all breast cancer tissues, and also at protein level, with a significant increase in its activity respect with normal breast tissue. Thus, Src acts as an important oncogenic switch linked to the activity of growth factors receptors, including EGFR and HER2 [63]. Indeed, Src activation is a critical event in the signaling downstream EGFR during breast cancer progression, stimulating migration and invasion of surrounding tissues [64]. Several preclinical studies have successfully used an Src inhibitor in order to counteract the aggressive phenotype of HER2+ breast cancer lines. However, also in the presence of controversial data, preclinical evidence suggests a role for SFKs regardless of HER2 expression. Src inhibitor PP2 reduced the S phase of cell cycles and inhibited colony formation, blocked cell migration/invasion and EMT in triple-negative breast cancer cell lines. Vimentin expression in this study was suggested as a potential biomarker to identify responsive cancer cell populations associated with an EMT phenotype [65]. The mesenchymal transition in breast cancer could be stimulated by different mechanisms, and it has been widely cited as an effective event associated with the promotion ability of Src in breast cancer. P-cadherin is frequently overexpressed in basal-like breast cancer, promoting cell invasion, stem cell activity, and tumorigenesis by the activation of Src signaling. Indeed, P-cadherin overexpression is significantly associated with Src activation in breast cancer cells, a molecular event that was also validated in a large series of primary tumor samples [66]. Also, leptin, one of the main adipokines secreted in breast tissue, can promote EMT through Src activation, leading to the secretion and activation of cell invasion and tumor progression [67].

Also, it has been observed that the over expression of an Src family member, Fyn, promoted the cell proliferation, migration, and invasion in breast cancer, whereas the Fyn depletion suppressed all these activities. Moreover, Fyn upregulated the expression of mesenchymal markers and EMT-related transcription factors, and downregulated the expression of epithelial markers. Furthermore, Fyn was transcriptionally regulated by FOXO1 and it mediated FGF2-induced EMT through both the PI3K/AKT and ERK/MAPK pathways [68].

Another member of SFKs, Lck, seems to be involved in breast cancer progression in association with Syk, a tyrosine kinase involved in many cellular processes including cell migration. Indeed, the cross-talk between Syk and Lck regulates hypoxia/reoxygenation, a process modelling breast cancer cell phenotype in advanced stages [69]. Since the involvement of different SFKs in breast cancer, different authors evaluated the effect of specific SFK inhibitors or knockdown of individual SFK members in breast cancer cells and compared the potential effects of pan-SFK and SFK-selective inhibitors on the formation of breast cancer metastasis in preclinical models [70,71].

In particular, Tabaries evaluated the effect of several pan-SFK and SFK-selective inhibitors or knockdown of individual SFK members on the formation of breast cancer liver metastases, concluding that pan-SFK inhibitors can enhance breast cancer metastasis to the liver. Also, the authors studied the expression of claudin-2, an important positive modulator of breast cancer liver metastasis, showing that the knockdown of individual SFK members, including Yes and Fyn, induced Claudin-2 expression, while contrary to this, Lyn-selective kinase inhibitor, bafetinib (INNO-406), reduced Claudin-2 expression and suppressed breast cancer liver metastasis [72].

### 4.2. Lung Cancer

In lung cancer, SFK overexpression has important implications in response of a variety of growth factors and in the modulation of their downstream effectors. EGFR inhibitors, like gefitinib and erlotinib, were found in non-small cell lung cancer (NSCLC) to reduce activation of Src and its substrates, suggesting the prominent role of Src in NSCLC progression. Indeed, some authors showed how, in combination with established therapeutic agents, AC-93253 iodide, a Src-EGFR crosstalk inhibitor, blocked cancer cell growth and motility [73]. In EGFR mutated NSCLC, EMT has been associated with acquired resistance to the EGFR inhibitor erlotinib. Moreover, “EGFR-addicted” cancer cell lines, when induced to undergo EMT, become erlotinib-resistant in vitro. To identify potential therapeutic vulnerabilities specifically within these mesenchymal, erlotinib-resistant cells, Wilson screened several different therapeutic agents, and the most potent against the mesenchymal cells was the Src inhibitor, dasatinib. Analysis of the tyrosine phospho-proteome revealed that several kinases along the Src/Fak pathway were differentially phosphorylated in the mesenchymal cells, and siRNA depletion of different Src/Fak pathway components in these mesenchymal cells caused apoptosis. These findings revealed a novel role for Src/Fak pathway kinases in drug resistance and identify dasatinib as a potential therapeutic for treatment of erlotinib resistance associated with EMT [74]. Reversible activation of tyrosine kinases and protein tyrosine phosphatases have been implicated in lung cancer in the regulation of cancer cell migration and invasion, interacting on Src and dishevelled associated activator of morphogenesis (DAAM1), a formin-like protein involved in the regulation of actin cytoskeletal remodeling. PTPN3 inhibited Src activity and Src-mediated phosphorylation of Tyr652 on DAAM1. Reversible tyrosine phosphorylation of DAAM1 by Src and PTPN3 regulated actin dynamics and lung cancer invasiveness [75]. The characterization of new genetic alterations is essential to assign effective personalized therapies in NSCLC; furthermore, finding stratification biomarkers is essential for successful personalized therapies. In this direction, Garmendia et al. showed that molecular alterations of Yes can be found in a significant subset of patients with lung cancer and they demonstrated an association between these alterations and prognosis using Yes status as predictive biomarker [76]. Also, the same authors demonstrated that stable Yes overexpression in human NSCLC cell lines induced proliferation, increased tumor growth and metastasis, but conversely, genetic depletion of Yes using siRNAs or CRISPR/Cas9 in human NSCLC cell lines reduced proliferation, survival, and invasion in vitro and tumor growth and lung metastatic growth. Treatment with the Src inhibitor dasatinib inhibited lung cancer proliferation, invasion, and migration in vitro and growth of subcutaneous tumors. Garmendia and colleagues also showed that in vivo, dasatinib treatment decreased tumor volume in high Yes-expressing NSCLC cell lines and patient-derived xenograft tumors, whereas low Yes-expressing NSCLC cell lines and patient-derived xenograft tumors were more resistant to dasatinib treatment. These data and results from other studies suggest that Yes protein expression may predict which patients with NSCLC will respond to Src inhibitors [77].

### 4.3. Thyroid Cancer

In the thyroid cancer, it has been demonstrated that targeting Src results in inhibition of growth, invasion, and migration both in vitro and in vivo, which can be enhanced through the combined inhibition of Src and the MAPK pathway. The combined inhibition of Src and the MAPK pathway holds great promise for improving the overall survival of advanced thyroid cancer patients with Braf and Ras mutations, and activation of the PI3K pathway represents an important biomarker of response for patients treated with this therapy [78]. Src and Lyn were the predominant SFKs expressed in thyroid cancer cells and dasatinib exposure resulted in the inhibition of cancer cell growth and metastatic tumor progression in vivo. Interestingly, the sensitivity to dasatinib was correlated with pFak levels but not pSFKs levels. The inhibition of metastatic progression appeared dependent on the ability of dasatinib to block the growth in distant sites rather than the cancer cell dissemination, providing a rationale for treatment of patients with advanced disease [79]. It is to note that distant metastases to the bone represent one of the most frequent progression also for thyroid cancer, and this aspect could represent a further rationale for the use of Src inhibitors in this tumor.

### 4.4. Glioblastoma

Glioma cells express and utilize several members of SFKs, including Src, Fyn, Yes, and Lyn, and their effects do not seem always to be functionally redundant. Interestingly, the importance of each member was dependent on both the growth factors and the extracellular substrates present. This situation could partially explain the presence of contradictory data when using pan-SFK inhibitors. For glioma growth and migration on laminin it was described as a role for Fyn, Src, and Yes [80,81].

Knockdown of Src, Fyn, and Yes significantly reduced migration of LN229 cells on collagen, while Lyn knockdown had no effect. In contrast, when using a different cell line, SF767, in the same experimental conditions only Yes knockdown significantly reduced cancer cell migration [82]. The Wnt family of secreted glycoproteins mediates the proliferation, invasion, and migration of glioma cells through β-arrestin-dependent signaling [83]. Indeed, in glioblastoma multiforme, β-arrestin 1 was overexpressed and it contributes to a poorer outcome. Knockdown of β-arrestin 1 decreased the activity of Src, and suppression of Src signaling was critically involved in β-arrestin 1 silencing-mediated suppression of GBM malignancies [84].

### 4.5. Colorectal Cancer

Elevated kinase activities of SFKs, mainly Src and Yes, were frequently observed in colorectal cancer (CRC), as compared with the normal counterpart, and this has been associated with tumor progression, metastasis, and a poor clinical outcome.

In CRC cell lines, Src activity was repetitively associated with invadopodia formation and degradation of ECM. The involvement of Src in invadopodia formation in CRC was also associated with cancer adaptation to redox stress [85,86]. While the degradation of ECM through Src-induced invadopodia formation seems to be FAK-independent, further studies revealed that TRIB1-mediated migration and invasion of CRC cells required up-regulation of MMP-2 through the activation of FAK/Src and ERK pathway [87]. Inflammatory responses contribute to promoting CRC invasive capacity and it impacts negatively on patient survival. SFKs expression in CRC and cancer associated immune cells correlates with the local inflammatory response in patients with advanced CRC [88,89].

Also, the downregulation of microRNA (miR)-542-3p is tightly associated with tumor progression via Src-related oncogenic pathways. In Src-transformed fibroblasts and human cancer cells that overexpress Src, miR-542-3p is substantially downregulated, and the ectopic expression of miR-542-3p suppresses tumor growth. MiR-542-3p expression is downregulated by the activation of Src-related signaling molecules, including epidermal growth factor receptor, K-Ras, and Ras/Raf/mitogen-activated protein kinase/extracellular signal-regulated kinase [90].

Yes seems to play in CRC a nonredundant function with respect to Src, and in fact its deletion is specifically associated with a reduction of b-catenin expression and in turn of cell migration. Yes silencing also determined a reduction in the capacity to generate liver metastases in vivo [91]. In addition, CRC patients whose liver metastases exhibited Yes (but not Src) activity had reduced survival compared to patients exhibiting Src (but not Yes) activity [92].

### 4.6. Pancreatic Cancer

Protein analysis indicates that Src is the most important member of SFKs to be overexpressed in pancreatic adenocarcinomas. However, SFKs RNA analysis also showed that other members were detectable and silencing of Yes, Lyn, Fyn, and Frk was able, as for Src, to significantly reduce pancreatic cancer cell migration, although at different levels [8]. When mice were injected subcutaneously with Src-silenced clone or they were treated with dasatinib, in presence of wild-type tumors, tumor size was decreased, and incidence of metastases was significantly reduced compared with controls [93]. In fact, also in this tumor model the inhibition of Src was associated with reduced MMPs activity and invasiveness [94]. The importance of pharmacological blockade of Src kinase activity in suppressing metastatic spreading was confirmed by the demonstration that decreased expression of endogenous Src inhibited pancreatic cancer cell progression through an anchorage-independent manner.

An integrin-stimulated effect was also described for Src in pancreatic cancer cells. This signaling pathway required recruitment of Src to the β3 integrin cytoplasmic tail, leading to Src activation, p130Cas phosphorylation and tumor cell survival that was independent of cell adhesion or FAK activation [95].

### 4.7. Prostate Cancer

Prostate cancer develops from an androgen dependent disease, and in the late stages of its progression, frequently becomes insensitive to androgen deprivation. Androgen-dependency escape is thought to be caused by several mechanisms, one of which is the overexpression of growth factor receptors. Several prostate cancer cell lines express functional EGFR and share a common vulnerability to EGFR inhibition in their proliferative and migratory ability. In order to investigate the role of Src in EGF-induced cancer cell migration Angelucci et al. tested different Src inhibitors, reporting that they modulated cell morphology and adhesive capacity on different physiological substrates. The action of small molecule inhibitors appeared to involve, in parallel with Src inhibition, the down-modulation of the active forms of paxillin and extracellular signal-regulated kinase (ERK) [96]. Indeed, several other studies demonstrated the efficacy of different strategies aiming at blocking Src activity, alone or in combination, both in early or advanced prostate cancer cell models [97,98,99].

Delle Monache et al. evaluated the anti-tumoral effect of paclitaxel in combination with Src inhibition, in a hormone-insensitive prostate cancer cell model. In vivo, combination treatment dramatically reduced prostate cancer tumor growth with a relevant difference in the density of new blood vessels with respect to control and single treatments. This reduction was determined by a concomitant impairment of endothelial cell migration and of VEGF release by cancer cells. In addition, the combination treatment determined a significant reduction in ROS production and HIF-1 stabilization in prostate cancer cells in respect to single treatments [19]. As well as in other solid tumors, hypoxia has been shown to support prostate cancer progression. In a hypoxic environment, Src activity was essential in promoting metastatic functions such as cell migration, invasion, and clonogenic survival in prostate cancer [100]. Lyn seems to play a non-redundant role in prostate cancer and Park et al. suggested that Lyn is more important than Src in determining prostate cancer cell proliferation in primary tissue, while Src specific inhibition affects primarily cellular migration [101].

### 4.8. Other Cancers

Src and Lyn kinases were upregulated in half of gastric cancer tissues and they associate with grade and metastases [102]. Gastric cancer cell lines were responsive to several Src inhibitors, slowing down proliferation and migration. Okamoto et al. demonstrated that the subsets of gastric cancer cells defined by a response to Src or tyrosine-protein kinase Met (MET) inhibitors were distinct, suggesting the analysis of MET amplification in the selection of patients to be treated with Src inhibitors [103,104]. The prognostic relevance of miR-140-5p in gastric cancer was investigated and Yes was identified as a novel target of miR-140-5p in regulating tumor progression; miR-140-5p serves as a potential prognostic factor in patients with gastric cancer, and miR-140-5p-mediated Yes inhibition is a novel mechanism behind the suppressive effects of miR-140-5p in gastric cancer [105].

In human biliary tract cancer, Src has been studied as a therapeutic target testing bosutinib, an orally active Src/Abl kinase inhibitor, alone or in combination with cytotoxic agents. Bosutinib abrogated phosphorylation of Src and its downstream molecules, and significantly increased G1 cell-cycle arrest and apoptosis. Bosutinib significantly inhibited cell migration and invasion and decreased EMT markers. Bosutinib combined with gemcitabine or cisplatin showed synergistic antiproliferative and antimigratory effects. In addition, these combinations further inhibited phosphorylation of Src and its downstream molecules and decreased to a major extent EMT marker expression compared with bosutinib alone [106].

The current data suggest an increased expression and activity in skin cancer in respect to normal skin mainly for Src and Yes. Some evidence indicates that Src is expressed in highly aggressive skin tumors, while Yes is expressed more in squamous cancer carcinoma compared to other skin cancers [107]. Melanoma frequently shows elevated Src kinase activity, and in preclinical studies dasatinib efficiently inhibited melanoma cell migration and invasion [108]. Other authors confirmed in melanoma cells the prominent role of Src in migration and invasion respect to proliferation, showing an effective role in stimulating MMPs release and phosphorylation of Fak and p130CAS [109]. Other members of SFKs could be involved in specific steps of skin cancer progression. Kim et al. showed that the EGF-stimulated anchorage-independent growth of A431 human epidermoid carcinoma cells could be inhibited by blocking the kinase activity of Hck and Blk [110].

## 5. Clinical Trials in Advanced Solid Tumors

High hopes have been deserved to the clinical use of Src inhibitors in the last 10 years. Expectancies derived mainly from copious and encouraging preclinical data and also from the availability of potent small molecule inhibitors. As elaborated in the following sections, different inhibitors have been tested alone or in various combinations so far, and some more studies are ongoing. In interpreting results from clinical trials in advanced solid tumors we must first consider some background information. The majority of trials have been conducted in unselected patients, frequently with heavily pretreated cancers. This represents the worst scenario in evaluating a targeted drug, and it renders the monotherapy an ambitious aim. The absence of patients’ stratification is an unfavorable situation for all targeted drugs; however, in the case of Src inhibitor, this aspect is associated with a further adverse clinical aspect: the absence of an effective predictive markers for Src inhibitors, even if different studies have examined a long list of potential biomarkers [111,112]. Another puzzling aspect, partially linked to the previous one, is the frequent absence of any molecular evidence in drug action. The clearest demonstration in this matter is represented by the potential effect on bone metastasis, largely demonstrated in preclinical studies. Drugs able to target Src were predicted in vitro to have a significant inhibitory effect on osteoclast activity [113]. However, in some studies, the analysis of serum markers of bone turnover in breast cancer did not confirm significant activity of Src inhibitors in modulating either bone resorption or bone deposition in patients [114]. On the contrary, dasatinib produced in bone metastatic prostate cancer the significant reduction of urinary N-telopeptide (uNTx) levels in the majority of patients, in an additive way with bisphosphonates treatment [115]. Results from another trial in breast cancer metastatic patients, indicated that dasatinib may be more effective at targeting bone metastasis compared with visceral metastases. However, in this study, a significant reduction in NTx levels, was observed only in a small cohort of HER+ patients with elevated baseline NTx levels [116]. The same challenging aspect in detecting Src kinase inhibition was described in blood and cancer tissue from dasatinib-treated patients [117]. The reasons for the failure in individuating a surrogate biomarker could be attributed to different mechanisms: inefficacy drug concentration delivered to tissue; technical obstacles in determining the necessary quantitative measures; redundancy in function of SFK family; Src kinase crosstalk with other signaling pathways. This latter aspect is exacerbated by the absence of patient stratification, in fact, the Src-mediated pathway can act both in co-operation or cross-talk with other signaling pathways rendering difficult to find the best surrogate marker at a single patient level [118]. For example, basal expression of pSTAT3 may be independent of Src, explaining therapeutic resistance, and precluding a correct analysis of dasatinib in biomarker-unselected cohorts [119]. In the following sections we illustrate the main hints from clinical trials performed with Src inhibitors in advanced solid tumors.

### 5.1. Dasatinib

Dasatinib (BMS-354825, Sprycel) is an orally available small molecule Src inhibitor that originally received FDA approval for its effective inhibition of the BCR/ABL fusion protein, and it is currently used to treat chronic myelogenous leukemia and Philadelphia-positive acute lymphoblastic leukemia. Dasatinib has a low inhibition specificity and its potential targets comprise also other SFKs, including Lck, Fyn, and Yes, with IC50 < 1.0 nmol/L [120]. Preclinical data support the hypothesis that dasatinib could interfere with the course of metastatic solid cancer, and they have prompted its utilization in clinical trials in advanced solid tumors. Numerous results from phase I/II trials conducted in different cancer types and according to very different protocols have been published since 2009 (Table 2). All clinical trials have faced the unfavorable toxicity profile of dasatinib, that includes fatigue, anorexia, nausea, diarrhea, pleural and pericardial effusion, and dyspnea. Approximately 30% of the patients who initiated the drug at a dose of 70 mg twice daily required a dose reduction because of toxicity. To date, measured clinical efficacy has been modest or null. Partial responses and stable diseases have been described in many situations, however mainly in combination regimens and in selected patient subpopulations. Dasatinib has limited single-agent activity in unselected patients with metastatic cancer. In advanced breast cancer, the most promising results have been reported in hormone responsive (HR) patients. In HER2-negative bone-prevalent metastatic patients partial responses in bone were noted in 23% of patient (25 total), all with HR-positive cancers, in combination with zolendronic acid [116]. In a phase II study, the progression free survival (PFS) was evaluated in advanced estrogen-receptor positive breast cancer patients after disease progression on a non-steroidal aromatase inhibitor, treated with exemestane plus dasatanib vs exemestane plus placebo. Of 157 partecipants, 63 had symptomatic bone disease. PFS was not significantly different in the two arms, but in the group exemestane plus dasatinib there was a 3/49 partial response (vs 0/49) and 21/49 stable disease (vs 14/49) were measured (ClinicalTrials.gov Identifier: NCT00767520). On the contrary, in bone-predominant metastatic breast cancer patients, without any molecular selection, dasatinib alone failed to achieve significant benefit also considering multiple evaluation methods, including PFS, response evaluation criteria in solid tumors response rate, monitoring of MUC-1 antigens, and of circulating tumor cells [114]. In women with invasive metastatic breast cancer, refractory to taxane and/or anthracycline therapy, partial response was found in 35% of HR positive tumors and only 6% in HR negative tumors when treated in combination with capecitabine [111]. Recently it has been demonstrated that dasatinib can be safely combined with trastuzumab and paclitaxel in HER2+ metastatic breast cancer patients (83% HR positive) and it resulted in being active with an ORR of 79% [121]. Indeed, combining trastuzumab with dasatinib has a strong preclinical rationale, as suggested by the key role of Src in trastuzumab resistant cancer cell models [122]. A phase III trial has been conducted in 1522 prostate cancer patients with evidence of metastatic disease and castration-resistant phenotype (CRPC) in order to compare dasatinib in combination with docetaxel. According to results published in 2013, dasatinib failed in improving overall survival or significantly modifying median PFS or median time to PSA progression [123]. In parallel, dasatinib alone has demonstrated biological activity on bone lesions in CRPC patients with significant reduction in bone turnover markers in association with stable metastatic disease after 3 months in a percentage of patients higher than 40% [115,124]. Although several gene signatures predictive of dasatinib response have been reported in vitro, the clinical validation of these signatures has been unproductive. Predictors derived from experimental models involving available cell lines confirmed their limited role in clinical predictivity. Dynamic analysis in metastatic biopsy of pFak, pPaxillin, and pSRC, in breast cancer patients, failed to reveal informative data, also due to technical challenges associated with the quality of metastatic biopsy and IHC analysis [125]. However, neither complex gene expression analysis of three different gene signatures previously validated in vitro, has permitted to define tumors clinically sensitive to dasatinib as a single agent [112]. The analysis of mutated signature in key oncogenes could offer, although not definitive, new suggestions. In advanced melanoma patients, the Braf/NRas/Kit wild type cohort has a greater benefit from dasatinib with dacarbazine [126]. Other data confirmed a higher partial response in Kit+ melanoma as compared to Kit- melanoma; however, these studies failed in confirming an increased activity than other Kit inhibitors [127,128]. Beside the clinical activity observed in Kit+ tumors, attributable, almost partially, to the inhibitory capacity of dasatinib toward Kit kinase, limited results obtained in mutated Kit tumors, confirmed the minimal activity of dasatinib in unselected patients. Genetic signature could mainly consider those activating mutations that have been associated with Src signaling. According to this interpretation we can cite the significant response observed in NSCLC patients with activating EGFR mutations [129].

### 5.2. Saracatinib

Saracatinib (AZD0530) is an orally bioavailable aniline-quinazoline highly selective for non-receptor tyrosine kinases, including Src (IC50 = 2.7 nM), Yes (IC50 = 4 nM), Lck (IC50 < 4 nM), and Abl (IC50 = 30 nM) [154]. Different from dasatinib, adverse effects of saracatinib at dose up to 175 mg daily were, for the most part, easily managed. Saracatinib displayed modest efficacy as monotherapy in unselected patients as demonstrated in early phase I/II trials (Table 3) [154,155,156]. Partial response was observed only occasionally as monotherapy. This includes patients with platinum-pretreated advanced NSCLC, in which a case of significant tumor reduction was also observed. Therefore, it was hypothesized, also considering data from dasatinib, that there may be a subpopulation of patients with advanced NSCLC that can benefit from Src inhibition [157]. Unfortunately, also in this trial, authors were not able to individuate predictive markers, being the adopted analysis of pSrc by IHC scarcely informative. An early study designed to evaluate bone resorption markers in advanced malignancies, mainly breast and colon cancer, indicated that saracatinib was effective in inhibiting osteoclast activity and suggested the use in metastatic bone disease [158]. This evidence has not been repeated in CRPC patient where the majority of cases exhibited progression of bone lesions [159]. Combination with gemcitabine resulted, as for dasatinib, well tolerated but it did not improve efficacy compared to what would be expected from gemcitabine alone [160]. Saracatinib was given to most patients with acceptable toxicity in combination with paclitaxel, with the most significant efficacy observed in patient with platinum-resistant ovarian cancer [161]. However, following phase III clinical trial failed in demonstrating the efficacy of the combination with taxanes in relapsed (platinum-resistant) ovarian cancer compared with paclitaxel alone (ClinicalTrials.gov identifier: NCT01196741). Preliminary evidence of antitumor activity was seen in patients with heavily pretreated advanced solid tumors (mainly colorectal cancer) when treated with saracatinib plus VEGF inhibitor cediranib. In fact, more than one-third of patients had a decrease in tumor size from baseline [162]. Otherwise the addition of saracatinib to cediranib did not improve efficacy in metastatic clear-cell renal cancer. In this study Fak overexpression predicted survival to drug combination [163]. Saracatinib currently does not have any active clinical trials in solid cancer.

### 5.3. Bosutinib

Bosutinib (SKI-606) was generally well tolerated, with a safety profile different from that of dasatinib in a similar patient population, with predominantly gastrointestinal adverse effects [168,169,170]. The safety profile was confirmed also for the combination with capecitabine for advanced solid tumors with stable disease observed only in colorectal (9/14) and breast cancer (5/11) patients. In addition, bosutinib demonstrated a more restricted inhibition activity toward Src/Abl compared with dasatinib. The phase II study in pretreated patients with locally advanced or metastatic breast cancer achieved a stable disease (>24 months) in more than 20% of patients with few partial responses, all in the subset of patients with HR positive disease [169]. These results prompted to design trials using a combination with endocrine therapy in advanced breast cancers. However, the combination with letrozole or exemestane in HR+ disease, although bosutinib demonstrated activity as single agent, resulted in unfavorable risk–benefit profile with early termination of the studies [171,172]. Several trials, although the safety profile of bosutinib, demonstrated limited efficacy in unselected patients also in combination with other drugs and determined in some cases the premature interruption of the trial (Table 4). Combination with capecitabine determined at best stable disease lasting < 24 weeks in breast cancer and colorectal cancer patients [173], and another phase I/II trial in breast cancer with the same combination was prematurely discontinued in 2013 when in the first phase was observed a limited efficacy (PR = 6%) (ClinicalTrials.gov identifier NCT00959946). A study evaluating bosutinib-exemestane combination vs. exemestane alone in women with HR+ HER2− breast cancer was terminated in 2012 due to unfavorable risk–benefit ratio of the study treatment (ClinicalTrials.gov identifier: NCT00793546). It was generally concluded that further translational biomarker analyses were needed to better define predictive biomarkers for bosutinib. New phase I trials are currently active: combination with pemetrexed in advanced solid tumors, (ClinicalTrials.gov identifier: NCT03023319, 2020 recruiting); combination with palbocicilib and fulvestrant for HR+Her2- advanced breast cancer refractory to an aromatase Inhibitor and a Cdk4/6 inhibitor (ClinicalTrials.gov identifier: NCT03854903, 2020 recruiting).

## 6. New Src Inhibitors in Clinical Trials

AZD0424 is a potent (IC50 approximately 4 nM) orally available, inhibitor of Src and Abl kinases with additional activity against Yes and Lck. A phase I study was performed with AZD0424 in advanced solid tumors generating evidence of Src inhibition at doses ≥ 20 mg per day. However, patients experienced multiple adverse events, also severe. AZD0424 displayed no evidence of efficacy as monotherapy [174]. New multi-kinase inhibitors with low IC50 for Src kinase are inhibitors for kinases involved in specific signaling pathways and could have efficacy in selected tumor populations. TPX-0022 is a novel inhibitor of Met/Csf1R/Src (IC50 = 0.12 nM for Src), and it is currently tested in recruiting Phase I study in patients with advanced solid tumors harboring genetic alterations in Met (ClinicalTrials.gov identifier: NCT03993873). TPX-0046 is a dual inhibitor of Ret and Src. The concomitant inhibition of the Src and Ret kinases was suggested to counteract the signaling bypass resistance and therefore it could increase the therapeutic effect seen with Ret inhibitors. A phase I/II study of TPX-0046, in subjects with advanced solid tumors harboring Ret fusions or mutations, including NSCLC and medullary thyroid cancer, is currently recruiting (ClinicalTrials.gov identifier: NCT04161391).

## 7. Conclusions

Since its first description, Src activation has been linked to modulation of signaling pathways, allowing a fundamental oncogenic process, such as continuous proliferation and cellular invasion. In order to evaluate SFKs role in tumor progression, many in vitro and in vivo experiments have been conducted so far: in an elevated number of tumor models, the data appear very consistent in defining SFKs as effective therapeutic targets able to inhibit tumor progression towards more aggressive and metastatic stages. However, this knowledge has demonstrated to be insufficient in defining exactly the clinical criteria of eligibility for SFKs inhibitors, and new data are pending just from preclinical studies. In fact, available in vitro models have revealed to be largely inadequate in their predictive capacity, and new experimental strategies are needed in order to optimize information that should be translated in clinic. In this sense, an important aspect is the implementation of the environment condition in cell culture. SFKs activity has revealed to be very susceptible to composition of ECM and SFKs’ role in FA implicates a tight sensing of the adhesive substrates. It is to note that during tumor progression, ECM composition changes accordingly to tumor remodeling activity and the spreading to distant sites, and this changeable situation could impact heavily on SFK functions and thus on treatment efficacy. In addition, also the utilization of 3D culture could refine current data, best resembling the potential activity linked to the initial phases of secondary growth.

Another largely unexplored aspect that should be improved is the evaluation of the specific role of single SFKs. A relevant number of publications have indicated that SFKs other than Src could exert a non-redundant role in cancer progression, and sometimes, in a worrying manner, also an opposite role. Thus, a new detailed description of specific function belonging to each SFKs could be useful also in interpreting puzzling results from the pan-SFK inhibitors used in clinical trials.

A further challenge is particularly important to be addressed before SFKs inhibitors could be effectively used in clinic. To date, we do not know effective predictive biomarkers for Src inhibition in clinic, and this is because either suggested marker is technically difficult to detect or is subjected to extreme inter- and intra-tumoral heterogeneity. Indeed, the best clinical efficacy of Src inhibitors has been achieved in subpopulations of patients with specific molecular signatures. In order to overcome this problem, a likely strategy is the utilization of surrogate markers that can offer the possibility to monitor in tumor cells the status of downstream effectors of Src. This approach, alongside to permit the evaluation of the effective pharmacological action of the drug in tumor cells, could allow also to monitor at individual level the inhibition of a downstream signaling pathway associated with cancer progression, stating its effective dependence on Src activity.

## Figures and Tables

**Table 1:** Distribution of Src Family tyrosine Kinase (SFK) proteins in normal and solid tumor tissues according to Human Protein Atlas database available from http://www.proteinatlas.org.

Protein Kinase	Tissue Distribution	Solid Tumor Distribution	
		Level of Expression	Tumor (*)
Src	most	strong	cervical, head and neck, pancreatic, skin, urothelial
		moderate	colorectal, lung, stomach
		weak	carcinoid, cervical
Yes	most	moderate	most (>60% = breast, colorectal, head and neck, liver, ovarian, prostate, testis, thyroid, urothelial)
Frk	most	strong	thyroid
		weak to moderate	carcinoid, colorectal, endometrial, liver, melanoma, renal, urothelial
Lyn	most	moderate to strong	liver, stomach
		weak	carcinoid, head and neck, thyroid
Fyn	brain, endocrine tissues, female tissues, hematopoietic cells, liver	moderate	glioma
		weak	carcinoid, thyroid
Blk	hematopoietic cells, lung	moderate to strong	endometrial
Fgr	hematopoietic cells, lung	weak	Carcinoid, colorectal, renal, thyroid
Hck	hematopoietic cells, lung	strong	endometrial, lung, renal, stomach
		weak	carcinoid, glioma, liver, ovarian, pancreas, skin
Lck	hematopoietic cells	negative	none
Srms	gastrointestinal, male tissue (**)	strong	colorectal, ovarian, prostate
		moderate	most

* only positivity >20% cases are reported; ** based on mRNA expression.

**Table 2:** Main characteristics of clinical trials designed in advanced solid tumors for the treatment with dasatinib alone or in combination with other drugs. Efficacy is to be considered as best clinical response, and when available, the population with the best response is indicated. PR = partial response; SD = stable disease; PFS = progression free survival.

Cancer	Phase	Combination	Efficacy	Dose	Year of Publication	Ref.
Advanced solid tumors	I	crizotinib	PR = 2% SD = 5% (mainly sarcoma and prostate cancer)	140 mg daily	2018	[130]
	I	gemcitabine	PR = 25% (pancreatic cancer)	From 70 mg daily to 100 mg daily	2012	[131]
	I	cetuximab	SD = 43% (better PFS in patients with low baseline TGF-α levels)	100 mg, 150 mg, 200 mg daily	2012	[132]
	I	none	SD = 19%	100 mg, 150 mg, 200 mg daily	2011	[133]
	I	none	SD = 16%	35 to 160 mg twice daily	2009	[134]
Advanced Non-Small Cell Lung Cancer	II	none	No response	70 mg twice daily	2017	[135]
	I/II	erlotinib	PR = 15% (EGFR mutated population)	70 mg daily	2014	[129]
	II	erlotinib	PR = 0% (EGFR-mutant and acquired resistance to EGFR-Tirosine Kinase Inhibitors)	70 mg twice daily, 100 mg daily	2011	[136]
	I/II	erlotinib	PR = 7%	50 mg twice, or 70 mg twice daily, or 140 mg daily	2010	[137]
	II	none	SD = 21%	100 mg twice daily reduced to 100 mg + 50 mg daily	2010	[138]
Advanced pancreatic cancer	I	Erlotinib gemcitabine	SD = 69%	70 mg daily reduced to 50 mg daily	2018	[139]
	II	gemcitabine	No response	100 mg daily	2017	[140]
	II	none	SD = 29% Metastatic cases	100 mg twice daily reduced to 70 mg twice daily	2013	[141]
Advanced breast cancer	II	trastuzumab paclitaxel	PR = 69% SD = 10% (HER2 + metastatic cases)	100 mg daily	2019	[121]
	II	paclitaxel	CR = 3% PR = 20% (mainly ER+)	120 mg daily	2018	[142]
	I/II	zolendronic acid	CR + PR = 23% SD = 13%	From 70 mg twice daily to 100 mg daily	2016	[116]
	II	none	PR = 4% (bone metastases)	70 mg twice daily and 100 mg daily	2016	[114]
	I	capecitabine	PR = 24% SD = 32%	50 or 70 mg twice or 100 mg daily	2013	[111]
	II	none	PR = 4% PR = 100% (HER2 + ER+)	100 mg twice daily reduced to 70 mg twice daily	2011	[143]
	I	paclitaxel	PR = 31% SD = 29%	70–120 mg daily	2011	[144]
Imatinib-refractory metastatic Gastrointestinal Stromal Tumors	II	none	PR = 25% (PFS = 50% in pSRC+)	70 mg twice daily	2018	[145]
High-grade and refractory Advanced Sarcoma	Ib	ipilimumab	No response	from 70mg to 100 mg or 70 mg twice, daily	2017	[146]
	II	none	SD (6 months) = 10–12% (leiomyosarcoma, osteosarcoma, undifferentiated pleomorphic sarcoma)	100 mg daily	2016	[147]
Advanced ovarian cancer	I	paclitaxel carboplatin	PR = 25% SD = 50%	100, 120, 150 mg daily	2012	[148]
Metastatic colorectal cancer	Ib/II	FOLFOX chemiotherapy w and w/o cetuximab	SD = 47% PR = 20%	100, 150, 200 mg daily	2017	[149]
	I	capecitabine oxaliplatin bevacizumab	PR = 10% SD = 60%	50 mg twice or 70 mg daily	2013	[150]
Metastatic castration resistant prostate cancer	II	none	SD = 19% PR = 4%	70 mg twice reduced to 100 mg daily	2013	[117]
	III	docetaxel	No response	100 mg daily	2013	[123]
	II	none	SD (3 months) = 44% SD (6 months) = 17%	100 mg daily	2011	[124]
	II	none	SD (3 months) = 43% SD (6 months) = 19% (Mainly bone metastases)	100 mg twice daily and 50 mg twice daily	2009	[115]
Advanced melanoma	II	none	PR = 6% PR (in KIT-) = 100% (stage IV, Mucosal, acral, or vulvovaginal)	70 mg twice daily	2017	[127]
	I	dacarbazine	PR+SD = 62% (metastatic cases)	70 mg twice daily	2012	[126]
	II	none	PR = 6% (no response in patients with mutated KIT)	100 mg twice daily reduced to 70 mg twice daily	2011	[128]
Advanced squamous cell carcinoma	II	none	No response Intolerable toxicity in the majority of cases.	140 mg daily	2013	[151]
Metastatic Head and Neck Squamous Cell Carcinoma	II	cetuximab	SD = 36% (mainly in low serum IL6)	150 mg daily	2017	[152]
	I	with or without erlotinib	No benefit respect to erlotinib alone (PR in low pStat3)	100 mg daily	2017	[119]
	II	none	SD = 17%	100 mg twice daily reduced to 150 mg daily or 50 mg twice daily	2011	[153]

**Table 3:** Main characteristics of clinical trials designed in advanced solid tumors for the treatment with saracatinib alone or in combination with other drugs. Efficacy is to be considered as best clinical response, and when available, the population with the best response is indicated. PR = partial response; SD = stable disease.

Cancer	Phase	Combination	Efficacy	Dose	Year of Publication	Ref.
Advanced solid tumors	I	none	SD = 25%	50, 125, 175 mg daily	2013	[155]
	I	cedinarib	SD = 63%	175 mg daily	2012	[162]
	I	paclitaxel (PTX) and/or carboplatin (CBP)	PR = 11% (PTX+CBP) PR = 21% (PTX) SD ≤ 15%	125, 175, 225, 250, 300 mg daily	2012	[161]
Advanced pancreatic cancer	I/II	gemcitabine	SD (4 months) = 23% PR = 9%	175 mg daily	2012	[160]
Advanced NSCLC	II	none	PR = 5% SD = 11%	175 mg daily	2014	[157]
Advanced thymic tumors	II	none	SD = 43%	175 mg daily	2015	[164]
Advanced gastric or gastro Oesophageal Junction (GEJ) Adenocarcinoma	II	none	SD = 18%	175 mg daily	2012	[165]
Advanced CR prostate cancer	II	none	SD (2 months) = 26%	175 mg daily	2016	[159]
	II	none	SD (5 months) = 11%	175 mg daily	2009	[154]
Advanced breast cancer	II	none	No response (ER-metastatic cases)	175 mg daily	2011	[156]
Recurrent and metastatic HNSCC	II	none	No response	175 mg daily	2011	[166]
Metastatic Clear-Cell Renal Cancer	II	cediranib	PR = 15%	175 mg daily	2016	[163]
Metastatic melanoma	II	none	SD = 9%	175 mg daily	2013	[167]
Relapsed ovarian cancer	III	paclitaxel	SD = 42% vs. 97% (placebo)	175 mg daily	2015	ClinicalTrials.gov Identifier: NCT01196741

**Table 4:** Main characteristics of clinical trials designed in advanced solid tumors for the treatment with bosutinib alone or in combination with other drugs. Efficacy is to be considered as best clinical response, and when available, the population with the best response is indicated. PR = partial response; SD = stable disease.

Cancer	Phase	Combination	Efficacy	Dose	Year	Ref.
Advanced solid tumors	I	capecitabine	SD = 64% (colorectal cancer) SD = 45% (breast cancer)	300 mg daily	2014	[173]
	I	none	SD = 47% (NSCLC) SD = 29% (colorectal) SD = 22% (pancreas cancer)	Escalating from 600 mg daily	2012	[168]
	I	none	SD = 12%	50 to 600 mg daily	2007	[170]
Advanced breast cancer	II	exemestane	PR = 2% SD = 7% (ER+ HER2-)	400 mg or 300 mg daily	2014	[172]
	II	letrozole	PR = 6% SD = 6% (ER+HER2-)	400 mg daily	2014	[171]
	II	none	PR = 5.5% SD = 32.9% SD = 41.7% (HER2+)	400 mg daily	2012	[169]
